# Integrating digital pathology with transcriptomic and epigenomic tools for predicting metastatic uterine tumor aggressiveness

**DOI:** 10.3389/fcell.2022.1052098

**Published:** 2022-11-18

**Authors:** Giorgia Sonzini, Sofia Granados-Aparici, Sabina Sanegre, Angel Diaz-Lagares, Juan Diaz-Martin, Carlos de Andrea, Núria Eritja, Aida Bao-Caamano, Nicolás Costa-Fraga, David García-Ros, Carmen Salguero-Aranda, Ben Davidson, Rafael López-López, Ignacio Melero, Samuel Navarro, Santiago Ramon y Cajal, Enrique de Alava, Xavier Matias-Guiu, Rosa Noguera

**Affiliations:** ^1^ Department of Pathology, Medical School, University of Valencia-INCLIVA, Valencia, Spain; ^2^ Cancer CIBER (CIBERONC), Madrid, Spain; ^3^ Epigenomics Unit, Cancer Epigenomics, Translational Medical Oncology Group (Oncomet), Health Research Institute of Santiago (IDIS), University Clinical Hospital of Santiago (CHUS/SERGAS), Santiago de Compostela, Spain; ^4^ Institute of Biomedicine of Sevilla (IBiS), Virgen del Rocio University Hospital/CSIC/University of Sevilla, Seville, Spain; ^5^ Clínica Universidad de Navarra, University of Navarra, Pamplona, Spain; ^6^ Institut de Recerca Biomèdica de LLeida (IRBLLEIDA), Institut d'Investigació Biomèdica de Bellvitge (IDIBELL), Department of Pathology, Hospital U Arnau de Vilanova and Hospital U de Bellvitge, University of Lleida - University of Barcelona, Barcelona, Spain; ^7^ Universidad de Santiago de Compostela (USC), Santiago de Compostela, Spain; ^8^ Institute of Clinical Medicine, Faculty of Medicine, University of Oslo, Oslo, Norway; ^9^ Department of Pathology, Oslo University Hospital, Norwegian Radium Hospital, Oslo, Norway; ^10^ Roche-Chus Joint Unit, Translational Medical Oncology Group (Oncomet), Health Research Institute of Santiago (IDIS), Santiago de Compostela, Spain; ^11^ Department of Pathology, Vall d'Hebron University Hospital, Autonoma University of Barcelona, Barcelona, Spain

**Keywords:** uterine adenocarcinoma, uterine leiomyosarcoma, lung metastasis, digital pathology, invasive tumor front, multiplex immunofluorescence, transcriptomics, methylomics

## Abstract

The incidence of new cancer cases is expected to increase significantly in the future, posing a worldwide problem. In this regard, precision oncology and its diagnostic tools are essential for developing personalized cancer treatments. Digital pathology (DP) is a particularly key strategy to study the interactions of tumor cells and the tumor microenvironment (TME), which play a crucial role in tumor initiation, progression and metastasis. The purpose of this study was to integrate data on the digital patterns of reticulin fiber scaffolding and the immune cell infiltrate, transcriptomic and epigenetic profiles in aggressive uterine adenocarcinoma (uADC), uterine leiomyosarcoma (uLMS) and their respective lung metastases, with the aim of obtaining key TME biomarkers that can help improve metastatic prediction and shed light on potential therapeutic targets. Automatized algorithms were used to analyze reticulin fiber architecture and immune infiltration in colocalized regions of interest (ROIs) of 133 invasive tumor front (ITF), 89 tumor niches and 70 target tissues in a total of six paired samples of uADC and nine of uLMS. Microdissected tissue from the ITF was employed for transcriptomic and epigenetic studies in primary and metastatic tumors. Reticulin fiber scaffolding was characterized by a large and loose reticular fiber network in uADC, while dense bundles were found in uLMS. Notably, more similarities between reticulin fibers were observed in paired uLMS then paired uADCs. Transcriptomic and multiplex immunofluorescence-based immune profiling showed a higher abundance of T and B cells in primary tumor and in metastatic uADC than uLMS. Moreover, the epigenetic signature of paired samples in uADCs showed more differences than paired samples in uLMS. Some epigenetic variation was also found between the ITF of metastatic uADC and uLMS. Altogether, our data suggest a correlation between morphological and molecular changes at the ITF and the degree of aggressiveness. The use of DP tools for characterizing reticulin scaffolding and immune cell infiltration at the ITF in paired samples together with information provided by omics analyses in a large cohort will hopefully help validate novel biomarkers of tumor aggressiveness, develop new drugs and improve patient quality of life in a much more efficient way.

## Introduction

The premise of precision oncology is to develop treatments targeting the molecular characteristics of patient tumors, drawing upon extensive databases that curate genetic and molecular features gathered from genome-wide association studies and tumor-sequencing efforts (Schwartzberg et al., 2017). However, clinicians will be confronted with a vast amount of complex molecular information derived from omics, drug biomarker testing, digital pathology and electronic health records (Alami et al., 2020), intensifying the gap between technological capacity, clinical knowledge, and the ability to translate molecular and digital pathology data into effective medical decision-making (Tamborero et al., 2020). Fortunately, multidisciplinary tumor boards are playing a key role in addressing this disparity, *via* emerging frameworks utilizing automated data capture, interpretation and reporting systems, from which researchers may subsequently share and harness results (Tamborero et al., 2020).

Uterine endometrial adenocarcinoma (uADC) is the most common cancer of the female reproductive organs in the developed world. uADC can present an aggressive form in a substantial number of cases, leading to poor prognosis (Huvila et al., 2021). Among the routes of metastasis in uADC patients are intra-abdominal spread, lymph node involvement and distant organ metastasis, and although very rare, this last route has a dramatic impact on prognosis and survival in uADC patients. In this context, in a recent study the lung was reported as the most common distant metastatic organ in patients with uADC (Mao et al., 2020). Uterine leiomyosarcoma (uLMS) is a rare tumor, accounting for 1%–2% of all uterine malignancies. Although cancer in patients suffering from this disease is often confined to the uterus, or with locoregional spread to the pelvis, a high number of cases develop metastatic disease, particularly at the lung (Roberts, Aynardi and Chu, 2018).

The last few decades have witnessed growing interest in understanding the characteristics underlying tumor invasion, with research centered on type-specific new stroma generated at the site of active tumor invasion [the invasive tumor front, ITF] (Antonicelli et al., 2012). Stromatogenesis, the formation of new stroma at the ITF, disrupts the continuity of normal tissues, facilitating the invasion process. This newly formed tumor-associated stroma is composed of stromal cells and the extracellular matrix (ECM), which is biochemically distinct in composition (Sivridis, Giatromanolaki and Koukourakis, 2004; Sanegre et al., 2021). Specifically, the new ECM is characterized by increased deposition of fibrillar collagen and cross-links, leading to increased tissue stiffness and causing desmoplasia, typical of the tumor (Eble and Niland, 2019). The newly deposited collagen fibers are reoriented and remodeled to generate larger and stiffer collagen bundles that contribute to the distinctive features of the ECM surrounding the tumor (Paolillo and Schinelli, 2019).

Among the different fibrillar collagens, type III collagen constitutes the fundamental protein of the reticulin fibers. Reticulin fibers are thin fibers of less than 2 µm in diameter that originate primarily from mesenchymal cells and undifferentiated fibroblasts. As fibroblasts differentiate, production of fibers and also type I collagen is increased. Reticulin fibers form an extensive branched network, with a mesh-like pattern, forming part of many organs, mainly as part of the basement membrane in epithelial tissue, in the basal lamina of adipose tissue and in locations with high cellular content, such as the endocrine glands, liver and bone marrow (Ushiki, 2002). They are found in high abundance in several disorders and tumors such as Castleman disease (Carbone et al., 2021), myeloproliferative neoplasms (Gleitz, Benabid and Schneider, 2021) and aggressive primary uADC and uLMS (Sanegre et al., 2021). Under the microscope, reticular fibers are indistinguishable from the other types in conventional stains, but they can be observed stained black with Gomori’s silver impregnation method, a technique routinely employed for visualization and characterization in many pathology departments (Ushiki, 2002). Along with the other ECM components, reticular fibers undergo changes during tumor stromatogenesis that affect their biotensegrity and thus have a functional impact on TME elements, such as the migratory potential of tumoral cells or the infiltration capacity of immune cells. Therefore, histomorphometric characterization of reticular fibers using digital analysis tools may add value for precision diagnostics in cancer (Montironi et al., 2020).

Changes in tissue composition that favor the growth of overt metastases are collectively known as the premetastatic niche (Kai, Drain and Weaver, 2019) which represents an abnormal microenvironment supportive of tumor growth (Paolillo and Schinelli, 2019). Secreted proteins from the primary tumor, such as proinflammatory cytokines and exosomes, can both directly activate tissue resident cells to synthesize and remodel the ECM in distant tissues and also stimulate recruitment of ECM-modifying cell populations (Kai, Drain and Weaver, 2019). The lung is the second most frequent site of metastatic focus. Normal lungs possess anatomical and cellular features that act as a defensive barrier against foreign pathogens and particles. However, under specific conditions, such as inflammatory state, the lung microenvironment displays features that may support carcinogenesis. The TME contains tumor-infiltrating immune cells that are closely linked to clinical prognosis in cancer patients (Xiao and Yu, 2021). Although malignant cells are able to evade the immune response, promoting tumor progression, tumor-infiltrating immune cells often initiate an immune response that aims to eliminate cancer cells. In this regard, the immune contexture and T cell abundance, functional activity, and spatial distribution in the TME are crucial prognostic and predictive factors (Galli et al., 2020). B cells, which are involved in humoral immunity of the adaptive immune system, can infiltrate the TME at the ITF. In mouse models, B cells have been observed to have an antitumor role by increasing T-cell function (Zhang and Zhang, 2020).

With the emergence of precision oncology, automated analysis of an ever-growing number of omics-generated biomarkers and the development of deep learning digital pathology models for their measurement in spatial context, all require methodological approaches for integrating the resulting data, which will bring insight into the molecular mechanisms leading to different pathways of original and metastatic tumor characterization in each patient (Mungenast et al., 2021). We recently characterized and compared the ITF of aggressive primary uADC and uLMS in a multicenter study, finding that despite morphometric resemblance between reticular fibers and high presence of macrophages, some variance could be observed in other immune cell populations, as well as distinctive gene expression and cell adhesion-related DNA methylation signatures (Sanegre et al., 2021). In this study, therefore, we used the same digital analysis, transcriptomic and epigenomic tools to investigate whether these changes are maintained in metastatic uADC and uLMS samples, and also compared them with the primary tumors. Overall, this study aims to enhance prediction of metastatic lung behavior and pinpoint possible new diagnostic, prognostic and/or therapeutic targets, which together with expert pathologist supervision and validation, could optimize routine pathological tasks in the future.

## Materials and methods

### Patients and case selection

A total of 24 uADC, 11 uLMS, 6 uADC lung metastases and 9 uLMS lung metastases (LM-uADC and LM-uLMS) were included in the study. We used formalin-fixed and paraffin-embedded (FFPE) tissue samples from uADC and uLMS primary tumors (previously described in Sanegre et al. (2021), with a particular focus on paired cases (primary tumors with their respective lung metastases). Tumors were classified according to the latest WHO criteria and were surgically staged and graded following the International Federation of Gynecology and Obstetrics (FIGO) classification and staging systems. The study was approved by the local research ethics committee, and specific informed consent was given. Whole slide FFPE tissue sections of 5 μm of selected uADC, uLMS and their respective lung metastases were stained with H&E (hematoxylin and eosin) and examined by a centralized expert pathologist group to select representative areas to include in the study. Sections of FFPE tissue samples were scanned using a digital slide scanner [Pannoramic 250 Flash II 2.0 (3D Histec)] prior to and following microdissection. Whole sections including the ITF were used for morphometric analysis. Serial uADC and uLMS whole slides were used for histomorphometric analysis of reticulin fibers (Gomori’s stain) and multiplex immunofluorescence-based immune profiling. ITF regions were identified in stained tissue by extrapolation of previous H&E-selected regions. A 5 × 4 mm region of interest (ROI) for each sample was identified in the ITF-stained area. These 5 × 4 mm regions were used to establish a correlation between the results of transcriptomic, epigenomic and morphometric studies. To achieve in-depth characterization of the reticular fibers and immune infiltrate of the ITF, the 5 × 4 mm ROIs were further broken down into 1 × 1 mm ROIs, representing the following categories: 1) tumor (uADC or uLMS or LM-uADC or LM-uLMS with absence of target tissue), 2) target tissue (myometrium for primary tumors and lung tissue for the respective metastases, excluding tumor infiltrated target tissue from the analysis), and 3) balanced representation of the invasion front (containing approximately 50% tumor and 50% target tissue). Cases and ROIs in which the algorithm failed due to insufficient or excessive staining were excluded from analysis, as were samples with unsatisfactory segmentation. Supplementary Table S1 indicates the number of 5 × 4 mm and 1 × 1 mm ROIs of each case in Gomori and multiplex immunofluorescence stained tissue. The interface between tumor tissue and adjacent myometrium or lung tissue was microdissected under the microscope for primary tumors and metastatic tumors, respectively. Microdissected tissue from the ITF was employed for transcriptomic and epigenomic studies. The amount of ITF microdissected tissue varied from case to case, but the median width was 5 mm (±1.66 mm) and median length 15 mm (±4.91 mm).

### Histomorphometric analysis of reticulin fiber

The architecture of reticulin fibers stained using Gomori’s method was studied in uADC, uLMS, LM-uADC and LM-uLMS. All samples were digitalized with the whole-slide scanner Ventana iScan HT (Roche) at ×20 with a resolution of 0.46 μm/pixel. We used QuPath open-source digital pathology software for sample visualization and identification of ROIs (Bankhead et al., 2017). 1 × 1 mm ROIs were exported to ImageJ (Schneider, Rasband and Eliceiri, 2012), where they were saved as TIFF for image analysis. The advanced morphometric methodology selected for this study was Gomoripath, an algorithm based on a probabilistic method for automatic reticulin fiber segmentation, as described in previous studies (Sanegre et al., 2021). Fifteen morphometric parameters defining the histological organization of reticular fibers were calculated for each fiber detected and the mean for each sample was calculated. Area, width, height and perimeter were used to determine the size of the fibers, while shape was defined by roundness, aspect ratio, perimeter ratio, shape factor, vertices and fractal dimension of the fibers (Tadeo et al., 2016). An additional size parameter defined in this study was deformity, corresponding to the degree of spatial dispersion of reticulin fibers relative to its center. The algorithm also measured the stained area of the tissue analyzed (excluding holes and damaged tissue), allowing us to determine the number of fibers per mm^2^ (density) and the percentage of fiber-stained area [%SA] (considering the sum of the areas of all fibers). Means and standard deviations were calculated for each morphometric parameter of uADC, uLMS and their respective lung metastases ROIs, and statistical comparison between the different case studies was performed by Student’s *t*-Test.

### Thyroid transcription factor 1 quantification in LM-uADC and LM-uLMS

Immunostaining for TTF1 was obtained in LM-uADC and LM-uLMS using a Dako Omnis platform. Antigen retrieval was performed using Target Retrieval Solution High pH (Dako-Agilent) at 97° for 30 min prior to incubation with a mouse monoclonal anti-TTF1 antibody (clone: SPT24, Gennova) for 30 min. EnVisionTM Flex Mouse Linker (Dako) was added for 10 min followed by EnVision FLEX/HRP (Dako) for 20 min and incubation with diaminobenzidine (DAB) chromogen for 5 min. QuPath v0.2.3 open-source digital pathology software was used for TTF1 quantification. A positive cell detection algorithm was employed to detect every nucleus that expressed TTF1. The number of positive cell nuclei per mm^2^ (density) at the ITF regions (1 × 1 mm) was calculated for each ROI in LM-uADC and LM-uLMS (Supplementary Table S1) and statistical differences were obtained by Student’s *t*-Test.

### Multiplex immunofluorescence staining and tissue imaging

A validated and standardized multiplex immunofluorescence protocol was used to characterize immune cell infiltrate in 1 × 1 mm ROIs of LM-ADCs and LM-LMS. This multiplex panel enabled simultaneous visualization and quantification of several cellular markers in a single FFPE tissue section. Briefly, whole-slide tissue sections were deparaffinized and subjected to sequential rounds of antibody staining, as previously described (Sanegre et al., 2021). Antigen retrieval was performed using Dako PT-Link heat-induced antigen retrieval with low pH (pH6) or high pH (pH9) target retrieval solution (Dako). The antibody panel included a mouse monoclonal anti-CD68 antibody (clone: PG-M1, ready-to-use; Agilent), a rabbit polyclonal anti-CD3 antibody (IgG, ready-to-use; Agilent), a mouse monoclonal anti-CD8 antibody (clone: C8/144B, ready-to-use; Agilent), a mouse monoclonal anti-CD20 antibody (IgG2α, clone: L26, ready-to-use; Agilent), a mouse monoclonal anti-CD66b antibody (clone: 80H3, dilution: 1:200; LS-Bio, Seattle, WA, United States) and DAPI. Tyramide signal amplification (TSA) with fluorophores Opal 650, 540, 570, 690, and 520 was used for visualization of CD3, CD8, CD20, CD68, and CD66b, respectively. Thereafter, nuclei were counterstained with spectral DAPI (Akoya Bioscience). Sections were then mounted with Faramount Aqueous Mounting Medium (Dako). Multiplexed immunofluorescence slides were scanned on a Vectra-Polaris Automated Quantitative Pathology Imaging System (Akoya Biosciences). Spectral unmixing was performed using inForm software (version 2.4.8, Akoya Biosciences), as described (Abengozar-Muela et al., 2020). Image analysis was performed using QuPath and Fiji/ImageJ. Briefly, cells were segmented based on nuclear detection using the StarDist 2D algorithm. A random trees algorithm classifier was trained for each cell marker. Cells were then subclassified as CD68^+^, CD3^+^, CD8^+^, CD66b^+^, and CD20^+^ cells. CD4^+^ T-cells were defined as CD3^+^ CD8^−^. Cells negative for these markers were defined as “other cell types”. Measurements were calculated as cell densities (cells/mm^2^).

### Transcriptomic profiling

Total RNA for gene expression assays was prepared from 5 µm FFPE tissue sections of microdissected ITF from LM-uADC and LM-uLMS using the Agencourt FormaPure kit (A33341; Beckman Coulter, Indianapolis, IN, United States) and following the manufacturers’ instructions. RNA concentration was determined with Qubit 4 fluorometer and Qubit^®^ RNA HS Reagent (Thermo Fisher Scientific, Waltham, MA, United States). We selected RNA samples from lung metastasis that had passed quality control evaluation (uADC = 6, uLMS = 7). Transcriptomic profiling was performed as previously described (Sanegre et al., 2021) using HTG EdgeSeq Precision Immuno-Oncology Panel. HTG EdgeSeq host software performed the alignment of the FASTQ files to the probe list, then results were parsed, and the output obtained as a read counts matrix. The relative abundance of immune and stromal cellular content (TME score) and specific cell types were estimated by analyzing parsed data files using the 23 HTG EdgeSeq™ Reveal Immunophenotyping Signatures. The signatures were implemented in HTG EdgeSeq™ Reveal statistical analysis software (https://www.htgmolecular.com/assets/htg/publications/HTG202015_WP-Immunopheno-04final_1.pdf), which uses xCell algorithm as a reference for gene enrichment analysis (Aran, Hu and Butte, 2017). Four of the 23 signatures measure stromal cells, including adipocytes, endothelial cells, epithelial cells, and cancer-associated fibroblasts. The remaining 19 signatures measure the relative abundance of different immune cells, including different subtypes of T-lymphocytes, B-lymphocytes, phagocytes, and granulocytes. iDEP v0.951 (http://bioinformatics.sdstate.edu/idep/) was used for pathway analysis of expression values from RNA-Seq data. Expression data from ITF of primary tumors generated in our former study (Sanegre et al., 2021) were used for comparisons with the transcriptional profile of the lung metastatic samples.

### DNA methylation profiling by Infinium Methylation EPIC array

Total genomic DNA (gDNA) from 10-μm FFPE tissue sections of the ITF from primary tumors and LM-uADC (n = 6) and LM-uLMS (n = 9) was isolated using the AllPrep DNA/RNA FFPE Kit (Qiagen) according to the manufacturer’s instructions. DNA samples were quantified by fluorometry with the Qubit 1 × dsDNA HS (High-Sensitivity) Assay Kit (Thermo Fisher). The suitability of the DNA for FFPE restoration was tested following the Infinium HD FFPE QC Assay (Illumina). All DNA samples (100–250 ng) were selected for bisulfite conversion using the EZ DNA Methylation kit (Zymo Research). The FFPE restoration step was followed by Infinium HD FFPE methylation assay using Infinium MethylationEPIC BeadChips, which cover over 850,000 CpG sites along the human genome (Moran, Arribas and Esteller, 2016). Whole-genome amplification and hybridization were performed on the BeadChips followed by single-base extension and analysis on a HiScan (Illumina) to assess the methylation status of cytosines. Quality control and preprocessing of methylation data were carried out using the R package RnBeads (Assenov et al., 2014). First, the Greedycut algorithm was used to filter out probes and/or samples. Next, probes overlapping with single nucleotide polymorphisms and probes whose sequence maps to multiple genomic locations (cross-reactive) were removed. Raw intensities obtained in the array were normalized using the BMIQ method. For each CpG site we obtained a specific β value by calculating the ratio of fluorescent signal between methylated (M) probe relative to the sum of the M and unmethylated (U) probes (β = M/(M + U)). β values ranged from 0 (no methylation) to 1 (completely methylated). Finally, hierarchical linear models were used to obtain between-group differences. *p* values were corrected for multiple testing (False Discovery Rate, FDR) using Benjamini–Hochberg method and a threshold of *p* < 0.05 was selected for significance. All statistical analyses were performed in the R statistical environment (v.3.6.1). Enrichment analysis of biological pathways for the methylation profiles was evaluated by gene ontology (GO) using Genecodis (Tabas-Madrid, Nogales-Cadenas and Pascual-Montano, 2012).

## Results

### ITFs and target tissues of uADC present a large and loose reticular network

**FIGURE 1:**
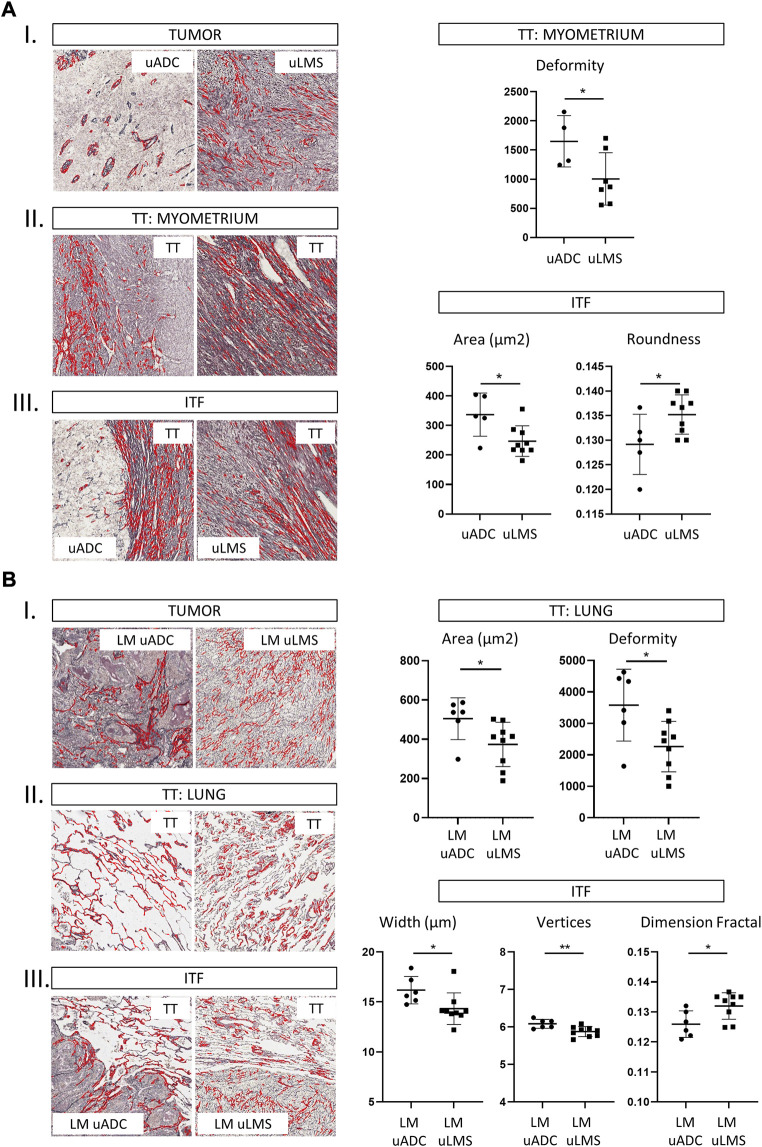
Histomorphometric characteristics of reticulin fibers between primary and metastatic tumors at the tumor area, ITF and target tissue in 1 × 1 mm ROIs. Representative images of reticulin fiber segmentation and statistically significant results of morphometric parameters. Reticulin fibers segmented using Gomori’s stain (in red) are shown in the histological images. Comparison between primary tumors metastasizing to lung **(A)** and lung metastasis from both uterine tumors **(B)**. Statistically significant differences in quantity, size and shape parameters of reticular fibers are shown (*p* values, * <0.05, **<0.01, ***<0.001). a. T, tumor, b. TT, target tissue and c. ITF, invasive tumor front. LM, lung metastasis.

We first studied the histological architecture of reticulin fibers in the tumor, myometrium and ITF, including more primary uADC and uLMS ROIs metastasizing to lung than were selected in Sanegre et al., 2021 to increase statistical robustness (Supplementary Table S1). Comparing reticulin scaffolding at the tumor area in uADC, reduced fiber deposition and multiple differences in fiber size and shape were observed relative to the myometrium and ITF, whose fibers did not differ significantly from each other (Table 1I). In contrast, the reticulin scaffolding in uLMS was similar in the three regions analyzed (Table 1II). We also investigated differences between uADC and uLMS. Comparison between tumor areas of uADC and uLMS showed decreased deposition of fibers in uADC, in addition to differences in size and shape parameters (Supplementary Table S2, I). Moreover, reticulin fibers of the myometrium and ITF regions showed a significant increase in size parameters (deformity and area, respectively) in uADC compared to uLMS, contrasting with a marked decrease in roundness at the ITF region in uADC (Figures 1AI−III). These results suggest that the fibers at the ITF region and myometrium of uADC have a greater extent in space and present a looser crosslinked network at the ITF region than those in uLMS.

**TABLE 1:** Comparison of histomorphometric characteristics of reticulin fibers between each tumor area in 1 × 1 mm ROIs.

	I			II.			III.			IV.		
	uADC			uLMS			LM uADC			LM uLMS		
	T vs*.* TT	T vs*.* ITF	TT vs*.* ITF	T vs*.* TT	T vs*.* ITF	TT vs*.* ITF	T vs*.* TT	T vs*.* ITF	TT vs*.* ITF	T vs*.* TT	T vs*.* ITF	TT vs*.* ITF
Quantity												
Fiber per mm2	TT**	ITF*						ITF*	ITF*			
%SA	TT*	ITF*										
Size												
Area	T*	T*						T*	TT*	TT**	ITF*	
Width	T**	T***							TT**	TT***		TT**
Height						ITF*				TT*	ITF*	
Perimeter								T*		TT**	ITF*	
Deformity	T*							T*	TT*	TT*		
Shape												
Roundness										T**	T**	
Aspect							T*					
Perimeter ratio										T***		
Shape				T_*_		ITF*						
Vertices	T**	T***					TT**		TT**	TT***	ITF**	TT***
Fractal dimension	T*	T**					TT*		TT*	TT**		TT*

Evaluation in primary uterine adenocarcinoma and uterine leiomyosarcoma metastasizing to lung (uADC and uLMS, respectively). Assessment in lung metastasis (LM uADC and LM uLMS, respectively). Comparisons between tumor (T), target tissue (TT) and invasive tumor front (ITF). Statistically significant differences in quantity, size and shape parameters of reticular fibers are shown (*p* values, * <0.05, **<0.01, ***<0.001) and the tissues with higher values are noted. LM: tumor lung metastasis; %SA: percentage of stained area. Area in μm^2^; width, length and perimeter in μm.

We next analyzed reticulin scaffolding in the three regions in each metastatic tumor. The tumor area of LM-uADC and LM-uLMS showed many differences in size and shape relative to their ITF and lung regions, as well as different ITF to the other regions in both metastases (Table 1I, II). Comparing reticulin scaffolding in the three regions between metastatic tumors revealed less fiber deposition at the tumor area in LM-uADC than LM-uLMS (Supplementary Table S2, II). Interestingly, reticulin fibers in the lung regions of LM-uADC showed higher deformity, as observed in the myometrium of the primary tumors, and increased area compared to those in LM-uLMS. Finally, significant differences in roundness, width and vertices were observed comparing reticulin fibers of ITF regions in LM-uADC and LM-uLMS (Figures 1BI−III). These results suggest that like the pattern observed in primary tumors, the reticulin fibers at the ITF are large and form a loose plexus in LM-uADC, whereas a dense fiber network is formed in LM-uLMS. To test whether these results were due to alveolar collapse with a difference in the number of TTF1-positive cells in LM-uLMS, we analyzed the percentage of cells expressing TTF1. No differences were observed between LM-uADC and LM-uLMS at the ITF regions (Supplementary Figure S1), suggesting that these findings could be due to reticulin fiber architectural changes without TTF1-related hyperplasia.

### ITFs and tumor areas of paired uLMS present reticulin fibers of similar size

**FIGURE 2:**
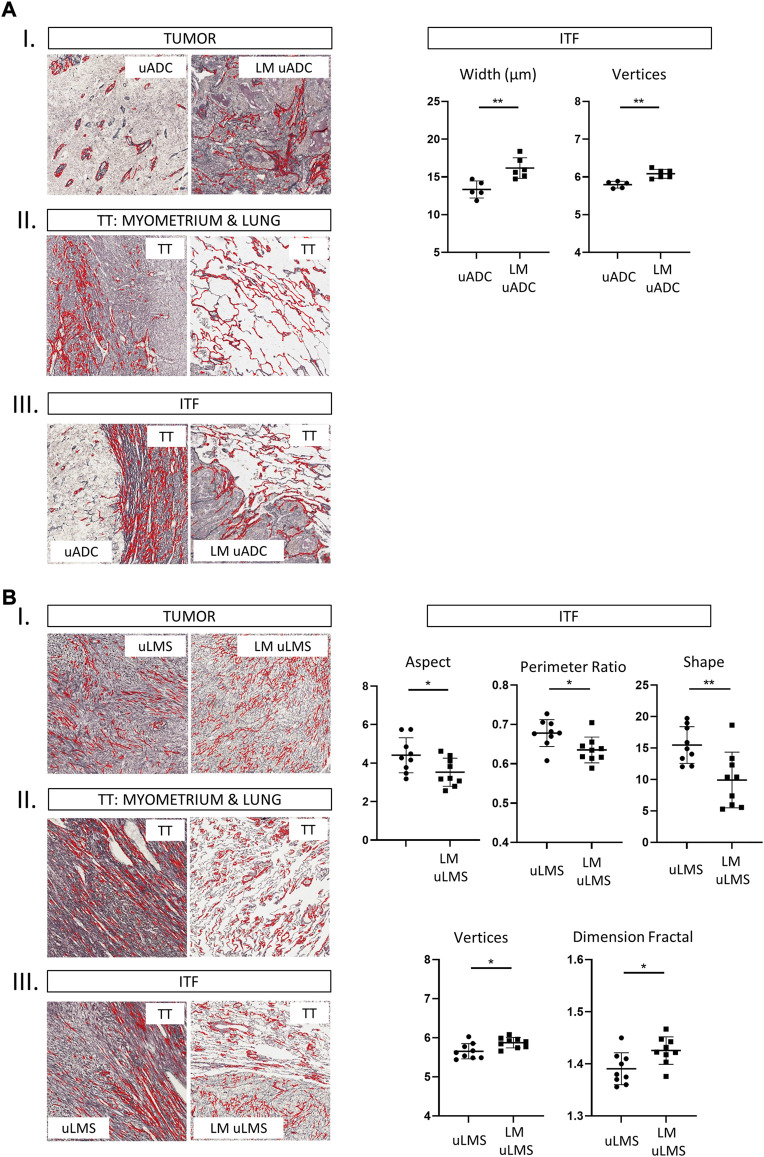
Histomorphometric characteristics of reticulin fibers from analysis of paired tumors at the tumor area, ITF and target tissue in 1 × 1 mm ROIs. Representative images of reticulin fiber segmentation and statistically significant results of morphometric parameters. Reticulin fibers segmented using Gomori’s stain (in red) are shown in the histological images. Comparison between primary uterine adenocarcinomas (uADC) and lung metastasis **(A)**, and primary uterine leiomyosarcoma (uLMS) and lung metastasis **(B)**. Statistically significant differences in quantity, size and shape parameters of reticular fibers are shown (*p* values, * <0.05, **<0.01, ***<0.001). a T, tumor, b. TT, target tissue and c. ITF, invasive tumor front. LM: lung metastasis.

We next compared paired tumors of uADC and uLMS (primary tumors with their respective lung metastases). Reticular scaffolding was different in parameters of size and shape between tumor areas in paired uADC, whereas no differences were observed in paired uLMS (Supplementary Table S2, III and IV). As expected, fibers in the target tissue (myometrium and lung regions) presented many size- and shape-related differences in both types of tumor (Supplementary Table S2, III and IV). Finally, comparing reticular fibers at the ITF region between paired uADC, width and vertices values were significant higher in LM-uADCs, suggesting that an even looser reticular network is formed in metastatic samples (Figures 2AI−III). When reticular fibers at the ITF region were compared in paired uLMS, no differences in size were observed, and significant differences in shape resulted in dense fiber bundles in LM-uLMS (Figures 2BI−III).

### ITFs in metastatic and paired tumors contain increased immune cell infiltrate in uADC

**FIGURE 3:**
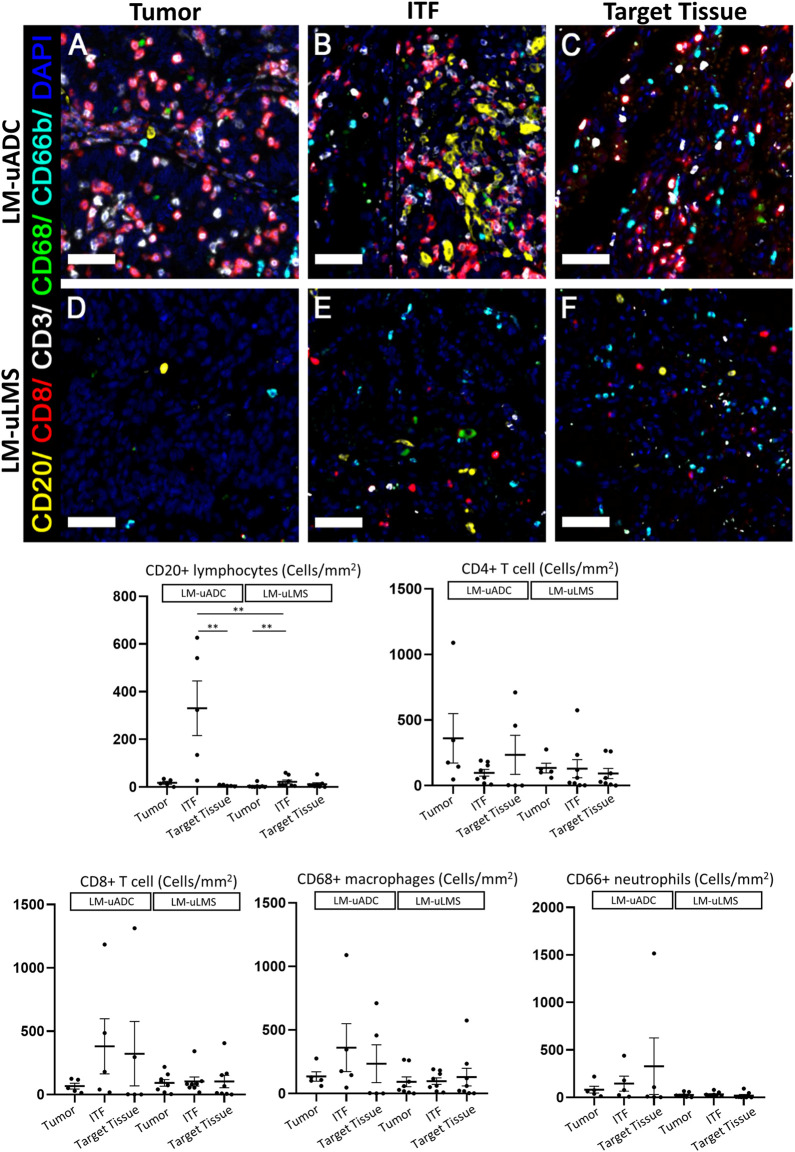
Immune infiltrate distribution in metastatic tumors. **(A,B, and C)** metastatic uterine adenocarcinoma) and **(D,E, and F)** metastatic uterine leiomyosarcoma). Representative multiplex immunofluorescence images in 1 × 1 mm ROIs in LM-uADC **(A–C)** and LM-uLMS **(D–F)**. Color code as follows: DAPI (blue), CD20 (yellow), CD68 (green), CD8 (red), CD3 (white), CD66 (cyan). Dot plots show the mean of the densities (number of cells/mm^2^) for each cell marker in each region for LM-uADC and LM-uLMS. *p* values and significance (^∗^ < 0.05, ^∗∗^ < 0.01) for statistical comparison between the means of LM-uADC and LM-uLMS at the tumor area, invasive front (ITF) and lung (TT) are displayed. Scale bars represent 50 µm.

Multiplex immunofluorescence was performed for CD20, CD3, CD8, CD66b, CD68 to estimate five different immune infiltrate subpopulations in LM-uADC and LM-uLMS. The 1 × 1 mm ROIs previously selected for reticulin fiber studies (annotated as tumor, target tissue or ITF) were used to estimate immune cell density. We observed diverse cellular compositions of the immune environments among the lesions (Figure 3). Within LM-uADC, higher densities of CD20^+^ B lymphocytes were found in ITF regions when compared to the tumor area and target tissue [*p* = 0.002] (Supplementary Table S3) which were more significant between the ITF and the target tissue [*p* = 0.0071] (Figure 3). No differences were found for the other immune cell infiltrates. Within LM-uLMS, higher densities of CD20^+^ B lymphocytes were also found in ITF regions when compared to the tumor area and target tissue [*p* = 0.012] (Supplementary Table S3). Similarly, in the LM-uLMS, a significantly higher infiltration of CD20^+^ B lymphocytes were found at the ITF when compared to the tumor area (*p* = 0.0089). No differences were found for the other immune cell infiltrates. When comparing the immune cell infiltrates of LM-uADC and LM-uLMS, higher infiltration of CD20^+^ B lymphocytes was found at the ITF of LM-uADC [*p* = 0.006] (Figure 3).

**FIGURE 4:**
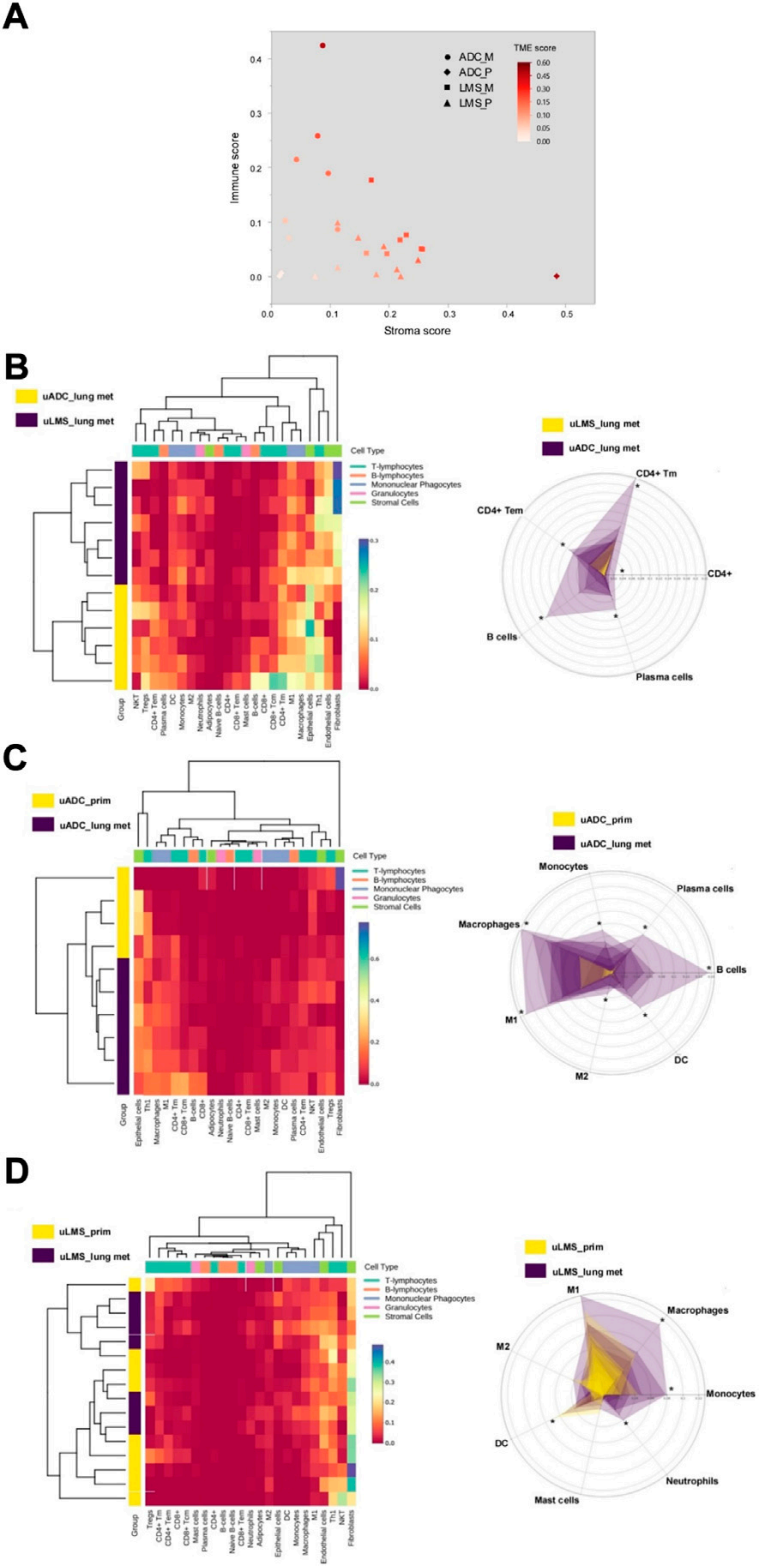
Evaluation of immunophenotyping signatures at the ITF of metastatic and paired tumors. **(A)** Estimation of immune and stromal cellular content. The TME (Tumor Microenvironment) score is the sum of the immune and stromal score **(B–D)** Left panels, hierarchical clustering of tumor samples and immune/stromal cell types based on the relative abundance score estimated with HTG EdgeSeq Reveal Immunophenotyping Signatures. Right panels, radar charts displaying the immunophenotyping signatures which showed the major differences in each comparison (*, denotes statistically significant differences in scores for relative abundance).

The immune and stroma scores of the ITF region in metastatic and primary tumors were obtained using expression data from the HTG EdgeSeq Precision Immuno-Oncology Panel (Figure 4A). In this region we observed higher immune scores for LM-uADC, whereas in LM-uLMS the stroma score was increased, possibly due to its intrinsic mesenchymal features. We next used the same expression data to determine the relative abundance of immune cell types. Regarding metastatic samples, major differences were observed in B cells/plasma cells, and CD4-T cell populations were also different, with increased abundance in LM-uADC compared with LM-uLMS metastasis (Figure 4B). We did not observe significant differences in CD8^+^ T cells (susceptible to exhaustion) in metastatic samples, and no notable differences were found between key T-cell exhaustion and activation markers (Supplementary Figure S2). We also performed the same analysis to compare the ITF of paired tumors of uADC and uLMS. We found a higher abundance of mononuclear phagocytes and B cells/plasma cells at the ITF of LM-uADC than that of primary uADC (Figure 4C), whereas the ITF of LM-uLMS were enriched in neutrophils, monocytes and macrophages and presented less abundance of dendritic cells than primary uLMS (Figure 4D).

### Transcriptomic profiling at the ITF reveals upregulation of genes involved in immune response in uADCs and double-strand break repair in uLMS

Differential expression analyses between ITF of LM-uADC and LM-uLMS identified 242 genes (FDR cutoff <0.1, log2-fold change ≥1). Interestingly, we found that antimicrobial peptides (*LCN2*, *DEFB1*) were upregulated at the ITF of LM-uADC, as was observed in primary tumors in our previous study (Sanegre et al., 2021) [Supplementary Table S4]. Both paired uLMS and uADC comparisons showed upregulation of lung markers in metastasis (*NKX2.1* (*TTF1*) and *AGER*), likely reflecting lung alveolar cells trapped in the tumoral mass (Supplementary Tables S5, S6).

**FIGURE 5:**
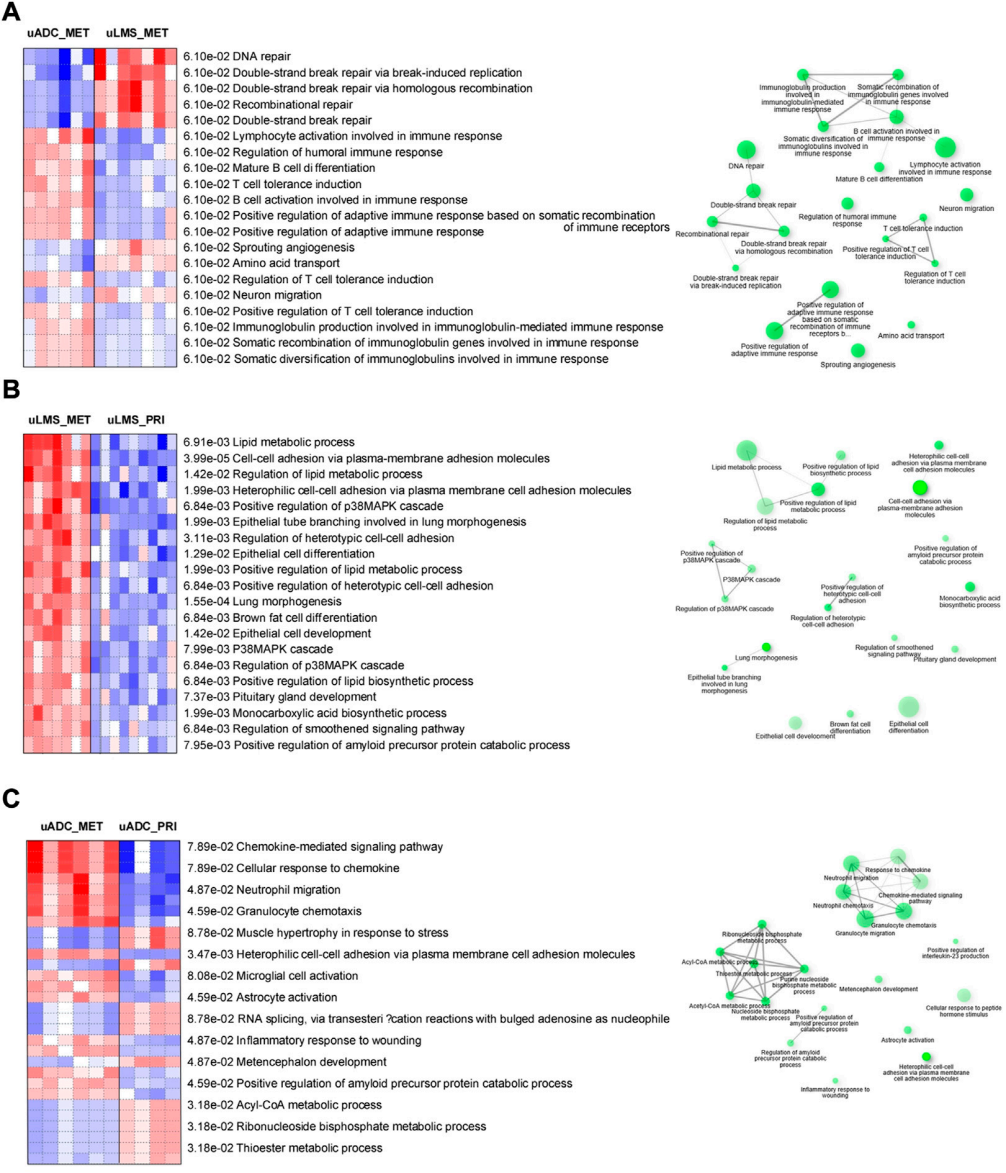
Pathway analyses of transcriptional profiling at the ITF of metastatic and paired tumors. Comparisons are indicated in each panel **(A–C)**. Left, red and blue indicate activated and suppressed GO cellular processes, respectively. Right, network of related pathways (nodes). Darker nodes are more significantly enriched gene sets. Bigger nodes represent larger gene sets. Thicker edges represent more overlapped genes.

Parametric gene set enrichment analysis using GO biological process terms showed lymphocyte activation and differentiation as well as adaptive immune response at the ITF of LM-uADC, in accordance with the higher relative abundance of B cells, plasma cells and CD4-T cells. Moreover, LM-uADC exhibited expression signatures related to somatic recombination of immunoglobulin genes. In contrast, the ITF of LM-uLMS exhibited upregulation of genes involved in double-strand break repair (Figure 5A). Pathway analysis of the ITF in paired uLMS denoted activation of several GO processes in metastasis such as lipid metabolism, MAPK cascade, cell-cell adhesion and lung morphogenesis (Figure 5B). When comparing the ITF of paired uADC, the most distinctive feature was the activation of granulocyte chemotaxis and migration in metastatic specimens (Figure 5C).

### Genome-wide DNA methylation analysis at the ITF shows a different epigenetic pattern in paired uADC

**FIGURE 6:**
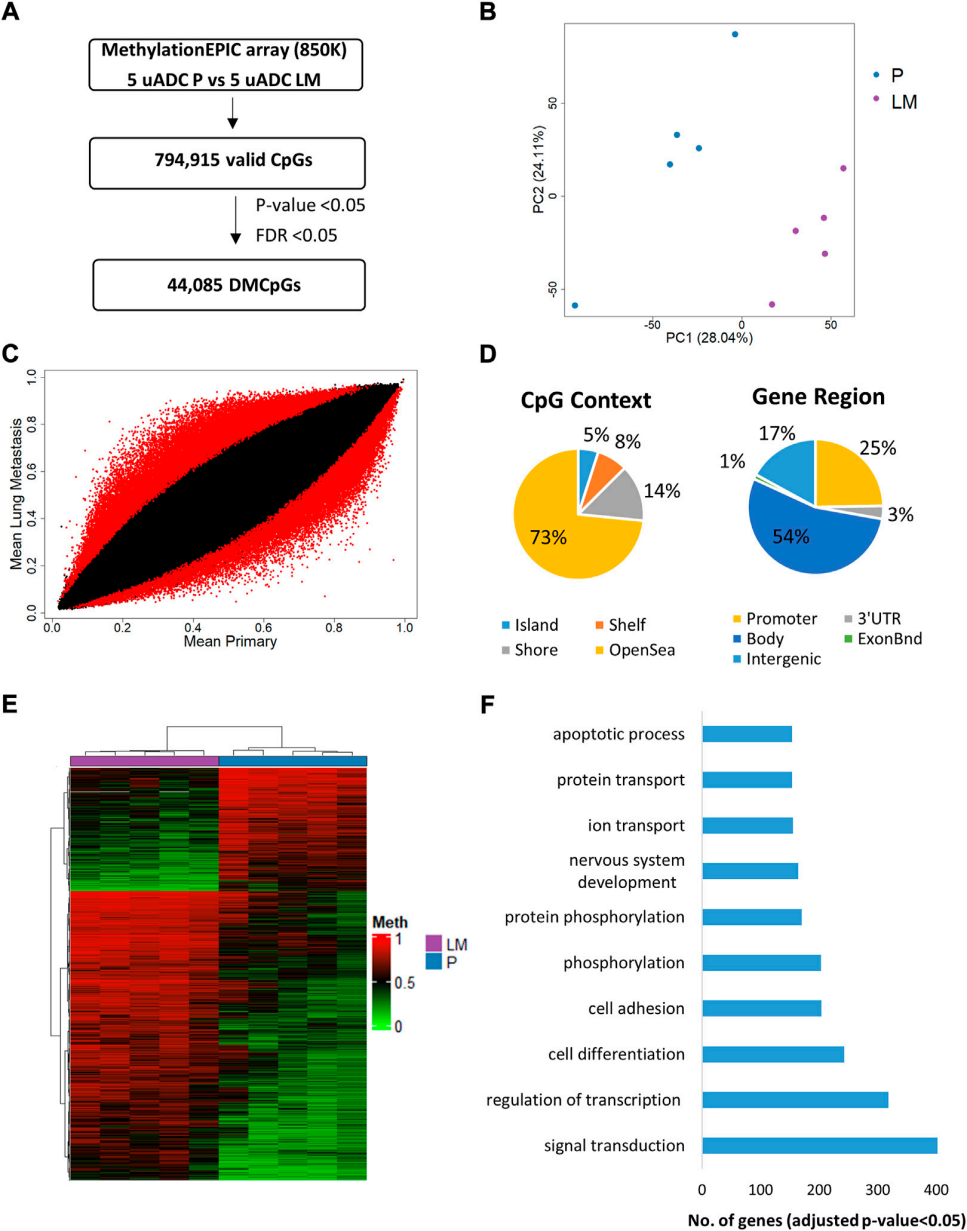
Genome-wide DNA methylation analysis of the ITF in paired tumors of uADC patients. **(A)** Schematic flowchart used to identify significant differentially methylated CpGs at the ITF. **(B)** Principal component analysis (PCA) of DNA methylation data at the ITF. **(C)** Scatter plot representing mean normalized levels of DNA methylation (β-values) at the ITF of primary tumors and lung metastasis. Dots in red show significant differentially methylated CpGs. **(D)** Genomic distribution of the 44,085 significant differentially methylated CpGs at the ITF by CpG context and gene region. **(E)** Hierarchical clustering of the 10,000 most differentially methylated CpGs (FDR <5%) in the ITF of primary and lung metastasis of uADC patients. **(F)** Gene ontology (GO) analysis of the biological process categories for the 10,000 most differentially methylated CpGs in the ITF of primary and lung metastasis of uADC. DMCpGs, differentially methylated CpGs; Meth, DNA methylation; P, Primary tumors; LM, lung metastasis, uADC, uterine adenocarcinomas.

The DNA methylation profile of the ITF in paired uADC patients was analyzed by Infinium Methylation EPIC array [EPIC array] (Figure 6A). Principal component analysis (PCA) showed a different methylation pattern in the ITF of paired tumors (Figure 6B). Importantly, we identified 44,085 significant differentially methylated CpGs (DMCpGs) (*p*-value < 0.05; FDR <0.05) between the ITF of paired tumors (DS). These DMCpGs were distributed across the genome, mainly in the body and promoter region of genes (Figure 6D). According to the density of CpGs, most differences were located in regions of DNA with low density of CpGs (open sea) and in CpG island (CGI) shore regions. Analysis of the 10,000 most identified DMCpGs in our study revealed an epigenetic signature able to clearly differentiate the ITF of paired tumors in uADC patients. GO analysis of this epigenetic signature showed enrichment of differentially methylated genes involved in relevant biological processes related to signal transduction, regulation of transcription, cell differentiation and cell adhesion, among others (Figure 6F). Table 2 shows the 25 most identified DMCpGs of the epigenetic signature that are located at CGIs or CGI shore regions of gene promoters.

**TABLE 2:** Top 25 significant differentially methylated CpGs from promoter CGIs/shore regions belonging to the epigenetic signature that differentiates the ITF of paired tumors in uADC patients.

TargetID^a^	Chr^b^	Position	Gene name	Accession number	Gene region	CpG density	Δβ^c^
cg10576280	10	124133822	PLEKHA1	NM_001001974	TSS1500	Shore	-0.65
cg02833127	4	177116733	SPATA4	NM_144644; NM_144644	1stExon; 5′UTR	Island	0.60
cg12307484	7	19158721	TWIST1	NM_000474	TSS1500	Shore	-0.57
cg21424940	7	19158666	TWIST1	NM_000474	TSS1500	Shore	-0.56
cg26250747	20	57225100	STX16	NM_001001433; NM_001134772; NM_003763; NM_001134773; NM_001204868NR_037943; NR_037942; NR_037941	TSS1500	Island	-0.56
cg02408153	2	177028655	HOXD3	NM_006898	TSS200	Shore	-0.56
cg03109841	17	73007443	ICT1	NM_001545; NM_001303265	TSS1500	Shore	0.55
cg14391419	7	19158647	TWIST1	NM_000474	TSS1500	Shore	-0.55
cg09258804	17	59532564	TBX4	NM_018488	TSS1500	Island	-0.52
cg03370588	15	52821290	MYO5A	NM_000259; NM_001142495	TSS20	Island	0.51
cg04481096	7	27142100	HOXA2	NM_006735	1stExon	Shore	-0.50
cg00886996	11	47599133	NDUFS3	NM_004551	TSS1500	Shore	0.50
cg16703737	11	47599085	NDUFS3	NM_004551	TSS1500	Shore	0.49
cg06653878	9	98080618	FANCC	NM_001243743; NM_001243744; NM_000136	TSS1500	Shore	0.49
cg07011913	2	177014626	HOXD4; MIR10B	NM_014621; NR_029609	TSS1500	Shore	-0.48
cg08717880	2	177014849	HOXD4; MIR10B	NM_014621; NR_029609	TSS1500; TSS200	Shore	-0.48
cg09386073	17	73007747	ICT1	NM_001545	TSS1500	Shore	0.48
cg10624122	7	19158747	TWIST1	NM_000474	TSS1500	Shore	-0.48
cg19692584	10	96161889	TBC1D12	NM_015188	TSS1500	Shore	0.48
cg06055873	7	27142204	HOXA2	NM_006735; NM_006735	1stExon; 5′UTR	Shore	-0.47
cg10126205	7	19158664	TWIST1	NM_000474	TSS1500	Shore	-0.47
cg22445940	20	57224919	STX16	NM_001134773; NM_003763; NM_001134772; NM_001001433	TSS1500	Island	-0.47
cg15543523	6	152127812	ESR1	NM_000125; NM_001122741; NM_001122740; NM_001122742	TSS1500; 5′UTR; TSS1500; 5′UTR	Shore	0.47
cg24736345	19	3093246	GNA11	NM_002067	TSS1500	Shore	0.47
cg09583532	2	177015999	HOXD4	NM_014621	TSS200	Shore	-0.46

^a^ Identification of CpG in EPIC array.

^b^ Chromosome.

^c^ Δβ-values (β-value LM - β-value P). A positive Δβ-value indicates hypermethylation in LM relative to P, and a negative Δβ-value indicates hypomethylation in LM relative to P. P, primary tumor; LM, lung metastasis. The CpGs are order according to the absolute Δβ-value. All the CpGs showed FDR <0.05.

**FIGURE 7:**
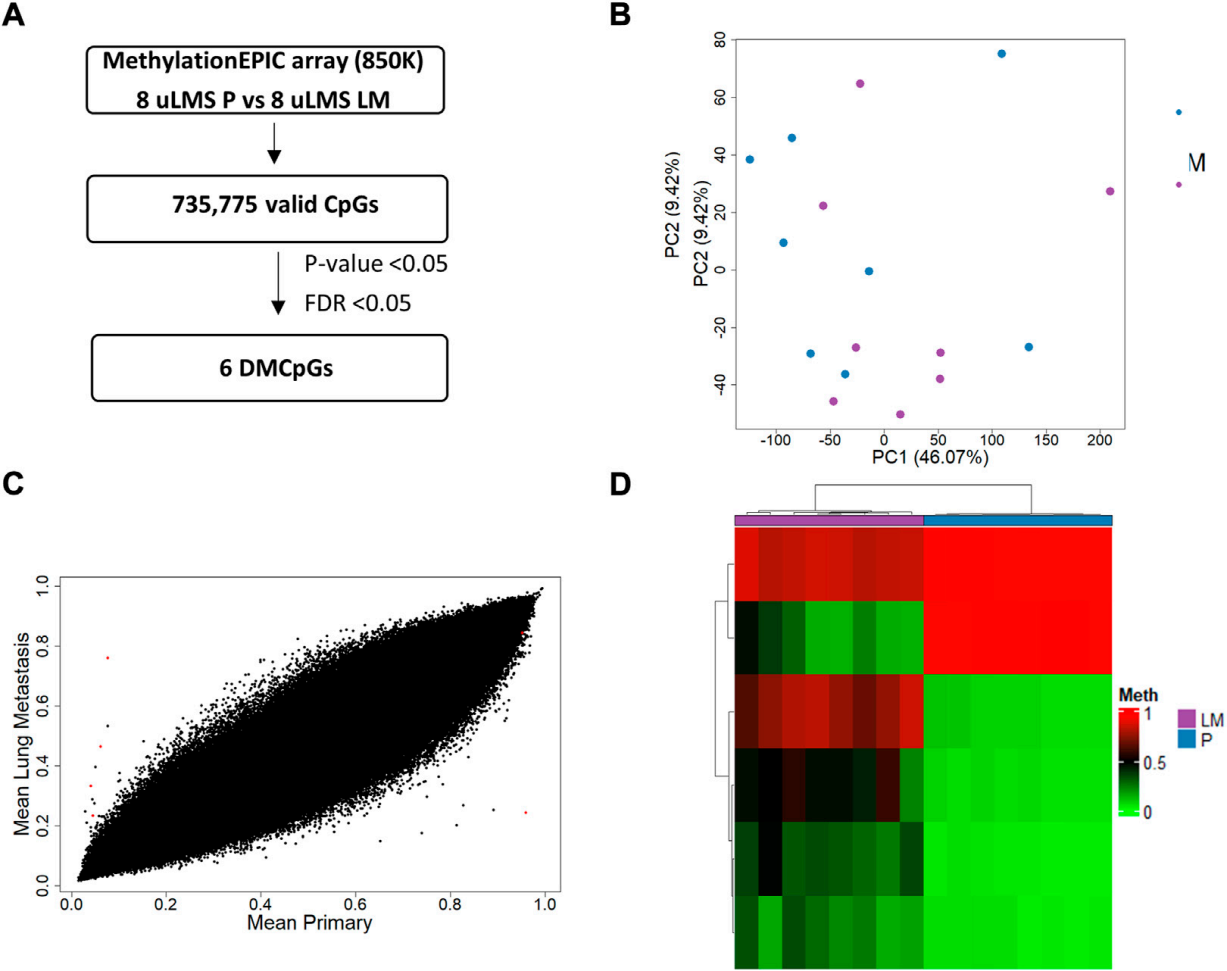
Genome-wide DNA methylation analysis of the ITF in paired tumors of uLMS patients. **(A)** Schematic flowchart used to identify significant differentially methylated CpGs at the ITF. **(B)** Principal component analysis (PCA) of DNA methylation data at the ITF. **(C)** Scatter plot representing mean normalized levels of DNA methylation (β-values) at the ITF of primary tumors and lung metastasis. Dots in red show significant differentially methylated CpGs. **(D)** Hierarchical clustering of the six differentially methylated CpGs (FDR <5%) found at the ITF of primary and lung metastasis of uLMS patients. DMCpGs, differentially methylated CpGs; Meth, DNA methylation; P, Primary tumors; LM, lung metastasis; uADC, uterine adenocarcinomas; uLMS, uterine leiomyosarcomas.

Next, we analyzed the DNA methylation profile of the ITF in paired tumors of uLMS patients (Figure 7A). The PCA showed that the paired tumors had a similar DNA methylation pattern at the ITF (Figure 7B). This concurs with the high similarity between the ITF of paired tumors showing the scatter plot in Figure 7C. Although the majority of CpGs analysed (735775 valid CpGs) were similar, we found an epigenetic signature of six significant DMCpGs (*p*-value < 0.05; FDR <0.05) which were able to differentiate the ITF of paired tumors in uLMS patients (Figure 7D). The six CpGs (cg03567028, cg02833127, cg03370588, cg03885684, cg04821107, cg00566642) of this epigenetic signature are indicated in Table 3.

**TABLE 3:** All identified significant differentially methylated CpGs of the epigenetic signature that differentiate the ITF of paired tumors in uLMS patients.


TargetID^a^	Chr^b^	Position	Gene name	Accession number	Gene region	CpG density	Δβ^c^
cg03567028	13	44544293			Intergenic	Island	-0.71
cg02833127	4	177116733	SPATA4	NM_144644; NM_144644	1stExon; 5′UTR	Island	0.69
cg03370588	15	52821290	MYO5A	NM_000259; NM_001142495	TSS200; TSS200	Island	0.40
cg03885684	2	120770471	EPB41L5	NM_020909	TSS200	Island	0.29
cg04821107	4	170947779	MFAP3L	NM_021647	TSS1500	Island	0.19
cg00566642	1	156825288	NTRK1; INSRR	NM_001007792; NM_014215	Body; Body	Shelf	-0.11

^a^ Identification of CpG in EPIC array.

^b^ Chromosome.

^c^ Δβ-values (β-value LM - β-value P). A positive Δβ-value indicates hypermethylation in LM relative to P, and a negative Δβ-value indicates hypomethylation in LM relative to P. P, primary tumor; LM, lung metastasis. The CpGs are ordered according to the absolute Δβ-value. All the CpGs showed FDR <0.05.

### ITFs of metastatic tumors exhibit some epigenetic differences

**FIGURE 8:**
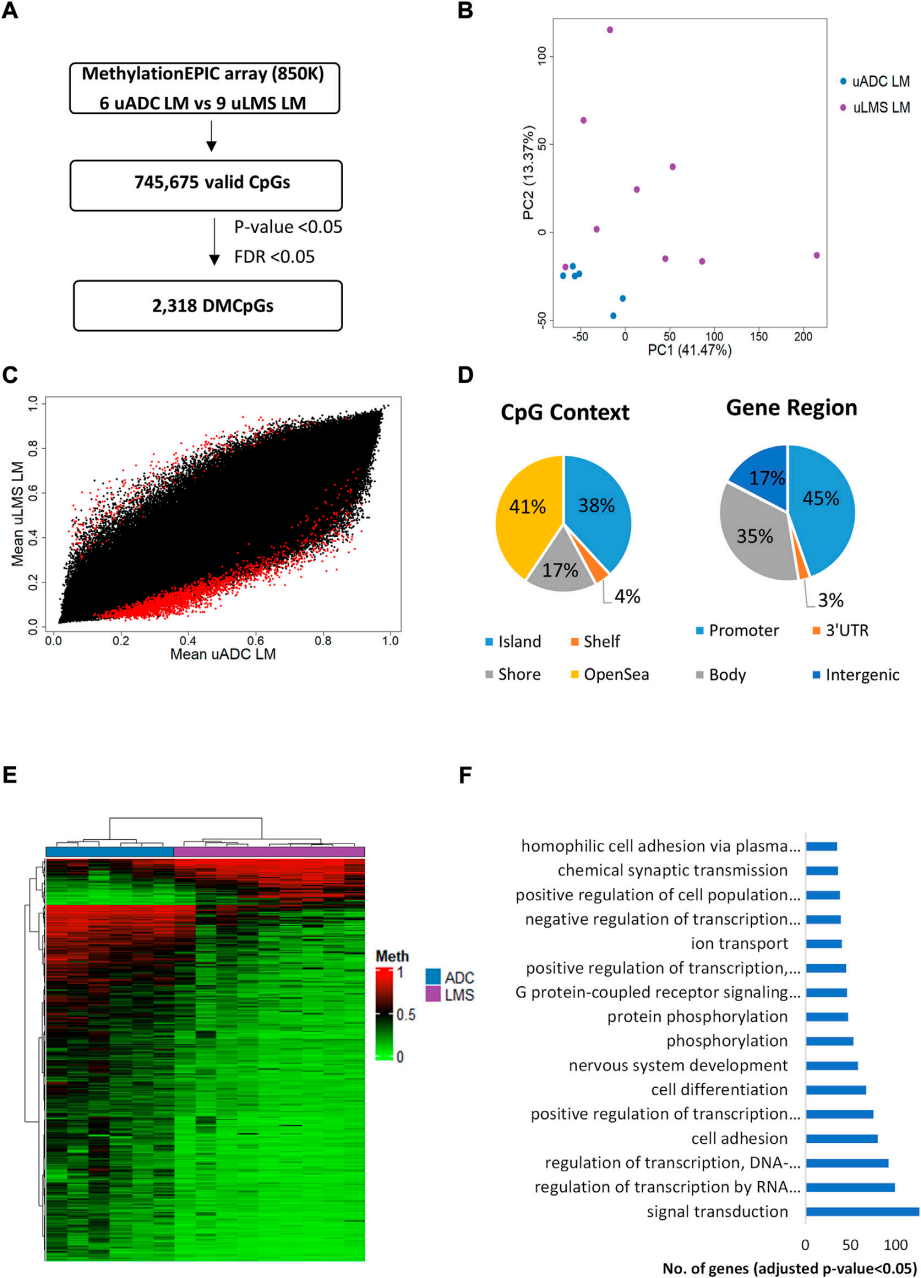
Genome-wide DNA methylation analysis of the ITF between metastatic tumors. **(A)** Schematic flowchart followed to obtain significant differentially methylated CpGs at the ITF. **(B)** Principal component analysis (PCA) of DNA methylation data at the ITF. **(C)** Scatter plot representing mean normalized levels of DNA methylation (β-values) at the ITF. Dots in red show significant differentially methylated CpGs. **(D)** Genomic distribution of the 2,318 significant differentially methylated CpGs in ITF by CpG context and gene region. **(E)** Hierarchical clustering of the 2,318 differentially methylated CpGs (FDR <5%) in the ITF of LM-uADC and LM-uLMS patients. **(F)** Gene ontology (GO) analysis of the biological process categories for the 2,318 differentially methylated CpGs at the ITF of LM-uADC and LM-uLMS. DMCpGs, differentially methylated CpGs; Meth, DNA methylation; LM, lung metastasis; uADC, uterine adenocarcinomas; uLMS, uterine leiomyosarcomas.

The DNA methylation pattern of the ITF was compared in LM-uADC and LM-uLMS patients using the EPIC array (Figure 8A). DNA methylation analysis by PCA showed some differences between metastases (Figure 8B). In particular, we identified 2,318 DMCpGs (*p*-value < 0.05; FDR <0.05) between the ITF of LM-uADC and LM-uLMS (Figures 8A–C), which were located mainly in the body and promoter region of genes (Figure 8D). Evaluating the density of CpGs, most differences were distributed between open sea regions and CGIs.

Most of the 2,318 observed DMCpGs defined an epigenetic signature that clearly differentiated the ITF of patients with LM-uADC vs LM-uLMS (Figure 8E). GO analysis of this epigenetic signature revealed enrichment of genes involved in important biological processes such as signal transduction, transcription regulation and cell adhesion, among others (Figure 8F). Table 4 shows the 25 most DMCpGs of the epigenetic signature found that are located at CGIs or CGI shore regions of gene promoters.

**TABLE 4:** Top 25 significant differentially methylated CpGs from promoter CGIs/shore regions belonging to the epigenetic signature that differentiates the ITF of lung metastatic uADC and uLMS.

TargetID^a^	Chr^b^	Position	Gene name	Accession number	Gene region	CpG density	Δβ^c^
cg20574490	chr8	102504666	GRHL2	NM_024915	TSS200	Island	-0.52
cg04060128	chr16	31488852	TGFB1I1	NM_001042454; NM_015927; NM_001164719	Body; Body; Body	Island	-0.50
cg19814116	chr1	6086448	KCNAB2	NM_172130; NM_003636; NM_003636; NM_172130	1stExon; 1stExon; 5′UTR; 5′UTR	Island	-0.49
cg11867697	chr12	122356394	WDR66	NM_144668; NM_001178003	TSS200; TSS200	Island	-0.49
cg03780851	chr8	102504564	GRHL2	NM_024915	TSS200	Island	-0.49
cg07637175	chr8	102504256	GRHL2	NM_024915	TSS1500	Shore	-0.48
cg05984244	chr16	31488238	TGFB1I1	NM_001042454; NM_015927; NM_001164719	Body; Body; Body	Island	-0.48
cg01970383	chr5	177729224	COL23A1	NM_173465	Body	Island	-0.48
cg02047049	chr9	35790836				Island	0.47
cg10191210	chr5	42992128				Island	-0.46
cg05230013	chr6	43212262	TTBK1	NM_032538	5′UTR	Shore	0.46
cg25658463	chr19	14543048	PKN1	NM_002741	TSS1500	Island	-0.45
cg01121275	chr5	115696282				Shore	0.45
cg07359545	chr22	19711327	GP1BB; SEPT5	NM_000407; NM_002688	Body; 3′UTR	Island	-0.45
cg26643631	chr8	102504903	GRHL2	NM_024915; NM_024915	5′UTR; 1stExon	Shore	-0.45
cg10207240	chr12	122356781	WDR66	NM_144668	5′UTR	Island	-0.44
cg18465694	chr22	36806185				Island	-0.43
cg26054842	chr7	155915712				Island	0.43
cg19769613	chr1	182028259	ZNF648	NM_001009992	5′UTR	Shore	0.43
cg17315219	chr19	48833559	EMP3	NM_001425	Body	Island	-0.43
cg12258785	chr3	24537407	THRB	NM_001128177; NM_001128176; NM_000461	TSS1500; TSS1500; TSS1500	Island	-0.42
cg14202477	chr8	102504859	GRHL2	NM_024915; NM_024915	1stExon; 5′UTR	Shore	-0.42
cg08334760	chr8	102504562	GRHL2	NM_024915	TSS200	Island	-0.42
cg14056864	chr3	146187210	PLSCR2	NM_001199978; NM_001199979; NM_020359	TSS200; Body; 5′UTR	Island	-0.42
cg26523005	chr3	42947267	ZNF662	NM_001134656; NM_207404	TSS1500; TSS200	Shore	0.42

^a^ Identification of CpG in EPIC array.

^b^ Chromosome.

^c^ Δβ-values (β-value LM-uADC - β-value LM-uLMS). A positive Δβ-value.

Indicates hypermethylation in LM-uADC relative to uLMS, and a negative Δβ-value indicates hypomethylation in LM-uADC relative. To LM-uLMS. LM, lung metastasis. The CpGs are ordered according to the absolute Δβ-value. All the CpGs showed FDR <0.05.

## Discussion

Critical for metastasis formation in distant organs, tumor invasion is the most important prognostic factor and the main actual cause of death in cancer patients (Kunita et al., 2018). Tumor cells that spread from the primary tumor are dependent on the TME, and presence of necessary stroma at secondary sites is critical in ensuring their activity and survival (Barcus and Longmore, 2021). In this study, we analyzed the ITF of uADC and uLMS paired tumors (uterine primary tumors and their respective lung metastases) based on the morphological parameters of the stroma component of the TME, reticulin fibers, and their transcriptomic and epigenetic profiles, looking for their potential predictive value in pulmonary metastases. Given the importance of immune cell infiltration in tumor invasion and cancer treatment, we also sought to characterize ITF-infiltrating immune cells at lung metastases.

Active invasion of cancer cells requires a process called stromatogenesis, defined as the formation of specific stroma at the ITF after peritumoral fibroblast proliferation (Giatromanolaki, Sivridis and Koukourakis, 2007). Tumor stroma consists of ECM, non-malignant cells and the signaling molecules they produce. It is an integral and vital component of primary tumors, evolving progressively as they develop, and is crucial in determining the growth characteristics, morphology, and invasion of the tumor (Sangaletti et al., 2017). The most abundant proteins within the ECM are fibrillar collagens I and III, whose quantity increases in many tumors (Barcus and Longmore, 2021). The presence of excess collagen I fibers and altered reticulin fiber architecture in the stromal microenvironment has been reported in oral squamous cell carcinoma, where differences were observed in quantity, distribution, and morphology at the ITF (Yinti et al., 2015). Another study in the same tumor type showed a change in length and thickness of reticulin fibers at the ITF, which correlated with degree of malignancy (Walke and Bhagat, 2017). Increased fiber deposition was observed at the ITF in uLMS compared to uADC, and these fibers always appeared smaller in size, suggesting less spatial expansion. Furthermore, the same results were obtained when the ITF of the respective lung metastases were compared without hyperplasia of type 2 pneumocytes. These differences could indicate organization of uLMS fibers into dense bundles. This linearized phenotype mirrors the pattern observed for collagen I fibers in breast cancer, which has been related to increased aggressiveness, and was accompanied by a dense cancer-associated ECM with aligned collagen highways (Amos and Choi, 2021) that tumor cells use to facilitate invasion (Cox and Erler, 2011). Moreover, second harmonic imaging microscopy of tissue and tumor slices revealed that collagen surrounding normal epithelia or early tumors is curly, smooth, and aligned parallel to the tumor surface, whereas invasive tumors present increased deposition of thickened, linearized, and arranged collagen fibers perpendicular to the tumor surface, increasing the stiffness of the ECM. These observed changes prompted the development of a prognostic tool named the tumor-associated collagen signature [TACS] (Provenzano et al., 2006). According to these and other studies (Conklin et al., 2011; Acerbi et al., 2015; Zhou et al., 2017; Jia et al., 2019; Seo et al., 2020), increased linearization and reorientation of reticulin fibers at the ITF in uLMS as compared to uADC could indicate higher degree of aggressiveness. These results contrast with the similarities previously found in the reticulin scaffolding at the ITFs in primary tumors, regardless of where they metastasized (Sanegre et al., 2021). The differences observed in the present study could therefore be related to the crucial role of the ECM in driving tumor progression at the primary site, differences that are only evident when the fate of lung metastases is considered. In addition, primary tumor-mediated ECM remodeling is implicated in the systematic appropriation of pre-metastatic niches (Cox and Erler, 2011), in which cells spread and colonize a secondary site, turning the TME into a mechanically complex niche due to changes in ECM rigidity. In morphometric study of reticulin scaffolding, the loose fiber plexus predominant in uADC could be associated with the pattern of ECM remodeling during the metastatic process deriving from its epithelial cell origin and the pulmonary niche. In contrast, the dense and linearized fiber bundle formation in uLMS may be due to their mesenchymal origin and subsequent development of a microenvironment with cellular and non-cellular components different to those found in the lung (Saggioro et al., 2020).

Besides its role in tumor invasion, collagen fibers from the TME affect T-cell differentiation and spatial distribution to modulate antitumorigenic immunity (Zhou et al., 2022). For example, the number of T cells present within a tumor and their ability to migrate and reach tumor cells are critical to achieving an effective antitumor response. It has been observed that in human lung and ovarian cancers, T cells preferentially accumulate in stromal regions that exhibit loose matrix architecture, rather than in dense regions. Therefore, remodeling and stiffening of the ECM and its components could be a barrier to T lymphocyte migration and infiltration (Nicolas-Boluda et al., 2021). Furthermore, aligned fibers in and around the perivascular regions of the tumor epithelial cells dictate the migratory trajectory of the T cells, preventing infiltration into the tumor. Consistently, a less dense matrix increased the ability of T cells to meet tumor cells, promoting immune infiltration into the tumor (Salmon et al., 2012). In the tumor cohort from this study, the results from the multiplex immunofluorescence-based immune profiling showed no differences in the spatial infiltration pattern of T cells, neutrophils, and macrophages. However, CD20^+^ B lymphocytes were found in significant numbers at the ITF, with a higher density in LM-uADC. Interestingly, an important role in the adaptive immune response is played by B lymphocytes, often characterized as a favorable prognostic biomarker in many cancer types. In fact, a study in colorectal cancer (CRC) revealed that patients with tumors highly infiltrated by CD20^+^ B lymphocytes were found to have prognostic advantage (Edin et al., 2019) and non-smoker patients with non-small-cell-lung cancer (NSCLC) showed better survival outcomes when tumors contained high number of tumor-infiltrating CD20^+^ B cells (Kinoshita et al., 2016). In line with the immune infiltration pattern, our findings from the transcriptomic analyses revealed increased B cell and CD4^+^ T cell populations and activation of adaptive immune response and lymphocytes at the ITF of LM-uADC in comparison with LM-uLMS. Indeed, activation of somatic recombination and diversification of immunoglobulins in ITF of LM-uADC probably reflect immunoglobulin production by B cells. When considering the morphometric parameters of reticulin fibers in the same context, these results may suggest a correlation between the selective accessibility of immune cells and reticulin fiber scaffolding. The large and isolated fibers with loose plexus at the ITFs in LM-uADCs could facilitate the passage of immune infiltrates such as CD4^+^ T and CD20^+^ B cells, whereas the dense bundled fiber network of LM-uLMS may hinder immune infiltration. In line with this hypothesis, greater immune infiltrates might be indicative of better prognosis in LM-uADC patients than in LM-uLMS patients.

Transcriptomic profiling revealed that antimicrobial peptides (*LCN2*, *DEFB1*) were upregulated at the ITF of LM-uADC compared to LM-uLMS, as reported at the ITF of primary tumors in our recent study (Sanegre et al., 2021). Moreover, activation of granulocyte chemotaxis and migration was also observed in LM-uADC, which may further potentiate this antimicrobial response. Thus, an impaired antimicrobial humoral response could be implicated in distant spread of uLMS. Interestingly, the ITF in LM-uLMS showed activation of DNA repair processes that could be a consequence of intense genotoxic treatment regimens. Furthermore, comparing activation of processes in uLMS metastasis vs. primary tumors, events such as lung morphogenesis, cell-cell adhesion and epithelial cell differentiation may correspond to the pulmonary microenvironment at the metastatic location.

Epigenetic profile analysis revealed more DNA methylation differences in paired samples in uADC compared to paired samples in uLMS. In addition, a different methylation profile was found at the ITF of LM-uADC and LM-uLMS. These variations in epigenetic profile were in partly related to changes in the methylation levels of genes involved in cell adhesion processes. These results are in line with findings from our previous study (Sanegre et al., 2021) where we observed a different methylation profile linked to cell adhesion in the ITF of primary tumors from uADC and uLMS patients. Cell adhesion is a biological process involved in maintaining the cell architecture (Ladoux and Mège, 2017) and regulating diverse homeostatic processes, including angiogenesis, migration and barrier function (Ebnet, 2017; Ford and Rajagopalan, 2018). In uterine neoplasms, regulation of cell adhesion processes has shown a key association with invasion/migration and with the immune/inflammatory milieu (Abal et al., 2007; Rutherford, Hill and Hopkins, 2018), indicating that the differences in methylation profile observed at the ITF of uADC and uLMS patients could contribute to explaining the differing aggressiveness of lung metastasis.

Our focus on the morphometric parameters of reticulin fibers revealed loose plexus scaffolding in LM-uADCs compared with the linearized fiber pattern in LM-uLMS. These differences, together with the increased quantity of immune cell infiltrates in LM-uADCs, suggest a lower degree of aggressiveness in LM-uADCs. In this context, LM-uADCs showed an increased methylation pattern of genes involved in cell adhesion, supporting the key role of this epigenetic mechanism in regulating the ECM and tumor immune milieu of LM-ADCs. Furthermore, transcriptomic analyses revealed increased immune infiltrate in LM-uADCs, corresponding mainly to B cell and CD4^+^ T cell lineages, with concomitant activation of an adaptive immune response. Finally, upregulation of antimicrobial response genes plus activation of chemotaxis could also contribute to better prognosis in LM-uADCs, as observed in our previous study. We therefore propose an integrative approach combining these methodologies as a prime target for analysis of tumorigenesis and development in primary and lung metastases at the ITF, which may improve precision diagnostics and therapeutics in patients suffering from aggressive uterine tumors.

## Data Availability

The datasets presented in this study can be found in online repositories. The names of the repository/repositories and accession number(s) can be found below. For primary tumors of uADC and uLMS, DNA methylation and expression profiling data are deposited with accession numbers GSE171142 (https://www.ncbi.nlm.nih.gov/geo/query/acc.cgi?acc=GSE171142) and GSE214779 (GSE214779 https://www.ncbi.nlm.nih.gov/geo/query/acc.cgi?acc=GSE214779), respectively. For metastatic tumors, accession numbers are GSE216313 (https://www.ncbi.nlm.nih.gov/geo/query/acc.cgi?acc=GSE216313) and GSE172043 (https://www.ncbi.nlm.nih.gov/geo/query/acc.cgi?acc=GSE172043), respectively.
