# Supplementary material for: Integrating digital pathology with transcriptomic and epigenomic tools for predicting metastatic uterine tumor aggressiveness
Source: Front Cell Dev Biol. 2022 Nov 18;10:1052098. doi: 10.3389/fcell.2022.1052098 (PMC9716026; doi:10.3389/fcell.2022.1052098)
Supplement: Supplementary file 3 [file Table1.docx]

|  | **Gomori** | | | | **Genomics** | **Epigenomics** |
| --- | --- | --- | --- | --- | --- | --- |
|  | 5x4 mm | 1x1 mm | | | 5x4 mm | 5x4 mm |
| **tumor ID** | **ITF** | **Tumor** | **Myometrium** | **ITF** | **ITF** | **ITF** |
| uADC-6 | Y | 3 | 3 | 3 | N | Y |
| uADC-28 | Y | 3 | 2 | 4 | Y | Y |
| uADC-29 | Y | 5 | 5 | 3 | Y | Y |
| uADC-30 | Y | N | N | N | Y | Y |
| uADC-38 | Y | 2 | 1 | 3 | Y | N |
| uADC-39 | Y | N | N | 6 | N | Y |
| **n tumors** | **6** | **4** | **4** | **5** | **4** | **5** |
| **n ROIs** | **6** | **13** | **11** | **19** | **4** | **5** |
|  |  |  |  |  |  |  |
|  | **Gomori** | | | | **Genomics** | **Epigenomics** |
|  | 5x4 mm | 1x1 mm | | | 5x4 mm | 5x4 mm |
| **tumor ID** | **ITF** | **Tumor** | **Myometrium** | **ITF** | **ITF** | **ITF** |
| uLMS-5 | Y | 3 | 1 | 3 | Y | Y |
| uLMS-7 | Y | 3 | 4 | 4 | Y | Y |
| uLMS-13 | N | 4 | N | 3 | Y | Y |
| uLMS-15 | Y | 3 | 2 | 3 | Y | Y |
| uLMS-17 | Y | 3 | 1 | 2 | Y | N |
| uLMS-19 | Y | 2 | 4 | 4 | Y | Y |
| uLMS-20 | Y | 4 | 2 | 5 | Y | Y |
| uLMS-21 | Y | 4 | 3 | 3 | Y | Y |
| uLMS-23 | Y | 2 | N | 4 | Y | Y |
| **n tumors** | **8** | **9** | **7** | **9** | **9** | **8** |
| **n ROIs** | **8** | **28** | **17** | **31** | **9** | **8** |

**Supplementary Table 1: Case selection and region of interest used in each characterization.**

|  | **Gomori** | | | | **Multiplex** | | | **Genomics** | **Epigenomics** | **TTF1** |
| --- | --- | --- | --- | --- | --- | --- | --- | --- | --- | --- |
|  | 5x4 mm | 1x1 mm | | | 1x1 mm | | | 5x4 mm | 5x4 mm | 1x1 mm |
| **tumor ID** | **ITF** | **Tumor** | **Lung** | **ITF** | **Tumor** | **Lung** | **ITF** | **ITF** | **ITF** | **ITF** |
| LM-uADC-6 | Y | 2 | 1 | 4 | 2 | 1 | 4 | Y | Y | 5 |
| LM-uADC-28 | Y | 4 | 4 | 2 | 4 | 4 | 2 | Y | Y | 2 |
| LM-uADC-29 | Y | 2 | 3 | 4 | 2 | 3 | 4 | Y | Y | N |
| LM-uADC-30 | Y | 2 | 4 | 3 | 2 | 4 | 3 | Y | Y | 2 |
| LM-uADC-38 | Y | 2 | 1 | 5 | 2 | 1 | 5 | Y | Y | 4 |
| LM-uADC-39 | Y | 5 | 2 | 3 | 5 | 2 | 3 | Y | Y | 4 |
| **n tumors** | **6** | **6** | **6** | **6** | **6** | **6** | **6** | **6** | **6** | **5** |
| **n ROIs** | **6** | **17** | **15** | **21** | **17** | **15** | **21** | **6** | **6** | **17** |
|  |  |  |  |  |  |  |  |  |  |  |
|  | **Gomori** |  |  |  | **Multiplex** | | | **Genomics** | **Epigenomics** | **TTF1** |
|  | 5x4 mm | 1x1 mm | | | 1x1 mm | | | 5x4 mm | 5x4 mm | 1x1 mm |
| **tumor ID** | **ITF** | **Tumor** | **Lung** | **ITF** | **Tumor** | **Lung** | **ITF** | **ITF** | **ITF** | **ITF** |
| LM-uLMS-5 | Y | 4 | 4 | 4 | 4 | 4 | 4 | Y | Y | 4 |
| LM-uLMS-7 | Y | 4 | 2 | 4 | 4 | 2 | 4 | Y | Y | 4 |
| LM-uLMS-13 | Y | 3 | 4 | 4 | 3 | 4 | 4 | N | Y | 2 |
| LM-uLMS-15 | Y | 3 | 4 | 4 | 3 | 4 | 4 | Y | Y | 4 |
| LM-uLMS-17 | Y | 3 | 3 | 4 | 3 | 3 | 4 | N | Y | 4 |
| LM-uLMS-19 | Y | 4 | 3 | 4 | 4 | 3 | 4 | Y | Y | 3 |
| LM-uLMS-20 | Y | 4 | 1 | 3 | 4 | 1 | 3 | Y | Y | 3 |
| LM-uLMS-21 | Y | 3 | 4 | 3 | 3 | 4 | 3 | Y | Y | 3 |
| LM-uLMS-23 | Y | 3 | 2 | 3 | 3 | 2 | 3 | Y | Y | 3 |
| **n tumors** | **9** | **9** | **9** | **9** | **9** | **9** | **9** | **7** | **9** | **9** |
| **n ROIs** | **9** | **31** | **27** | **33** | **31** | **27** | **33** | **7** | **9** | **30** |

The total numbers of region of interest (ROIs) and tumors used for the studies are shown. The number of ROIs are based on a previous study (Sanegre *et al.,* 2021) and have been increased in this study for primary tumor with metastasis in lung. ID: tumor identification; uADC: uterine adenocarcinoma; uLMS: uterine leiomyosarcoma, LM: lung metastasis; ITF: invasive tumor front; TTF1: Thyroid transcription factor 1.

# 
