# Supplementary material for: Integrating digital pathology with transcriptomic and epigenomic tools for predicting metastatic uterine tumor aggressiveness
Source: Front Cell Dev Biol. 2022 Nov 18;10:1052098. doi: 10.3389/fcell.2022.1052098 (PMC9716026; doi:10.3389/fcell.2022.1052098)
Supplement: Supplementary file 4 [file Table2.docx]

|  | I. | | | II. | | | III. | | | | IV. | | | |
| --- | --- | --- | --- | --- | --- | --- | --- | --- | --- | --- | --- | --- | --- | --- |
|  | **uADC**  **vs**  **uLMS** | | | **LM uADC**  **vs**  **LM uLMS** | | | **uADC**  **vs**  **LM uADC** | | | | **uLMS**  **vs**  **LM uLMS** | | | |
|  | T | TT | ITF | T | TT | ITF | T | TT | ITF | T | | TT | ITF |  |
| Quantity |  |  |  |  |  |  |  |  |  |  | |  |  |  |
| Fibers per mm2 | uLMS  *** |  |  | LM uLMS  * |  |  |  |  |  |  | |  |  |  |
| %SA | uLMS  ** |  |  |  |  |  |  |  |  |  | |  |  |  |
| Size |  |  |  |  |  |  |  |  |  |  | |  |  |  |
| Area | uADC  *** |  | uADC  * | LM uADC  ** | LM uADC  * |  |  | LM uADC  * |  |  | | LM uLMS  ** |  |  |
| Width | uADC  ** |  |  | LM uADC  ** |  | LM uADC  * |  | LM uADC  ** | LM uADC  ** |  | | LM uLMS  *** |  |  |
| Height |  |  |  | LM uADC  *** |  |  | LM uADC  * | LM uADC  * |  |  | |  |  |  |
| Perimeter |  |  |  | LM uADC  *** |  |  |  |  |  |  | | LM uLMS  ** |  |  |
| Deformity |  | uADC  * |  | LM uADC  *** | LM uADC  * |  |  | LM uADC  * |  |  | | LM uLMS  ** |  |  |
| Shape |  |  |  |  |  |  |  |  |  |  | |  |  |  |
| Roundness |  |  | uLMS  * | LM uLMS  *** |  | LM uLMS  * | uADC  ** |  |  |  | | uLMS  ** |  |  |
| Aspect | uLMS  * |  |  |  |  |  | LM uADC  ** |  |  |  | |  | uLMS  * |  |
| Perimeter ratio | uLMS  ** |  |  | LM uLMS  * |  |  |  |  |  |  | |  | uLMS  * |  |
| Shape |  |  |  |  |  |  |  |  |  |  | |  | uLMS  ** |  |
| Vertices | uADC  *** |  |  | LM uADC  *** |  | LM uADC  ** | uADC  * | LM uADC  *** | LM uADC  ** |  | | LM uLMS  *** | LM uLMS  * |  |
| Fractal dimension | uADC  *** |  |  |  |  |  | uADC  *** |  |  |  | | LM uLMS  ** | LM uLMS  * |  |

# Supplementary Table 2. Comparison of histomorphometric characteristics of reticulin fibers between primary, metastatic and paired tumors at the tumor area, ITF and target tissue in 1x1 mm ROIs.

# Comparison between primary tumors metastasizing to lung (I) and lung metastasis from both uterine tumors (II). Comparison between primary uterine adenocarcinomas (uADC) and lung metastasis (III), and primary uterine leiomyosarcoma (uLMS) and lung metastasis (IV). Statistically significant differences in quantity, size and shape parameters of reticular fibers are shown (p values, *<0.05, **<0.01, ***<0.001) and the tissues with higher values are noted. ITF: invasive tumor front; LM: lung metastasis; T: tumor; TT: target tissue; %SA: percentage of stained area. Area in μm^2^; width, length and perimeter in μm.
