# Supplementary material for: Integrating digital pathology with transcriptomic and epigenomic tools for predicting metastatic uterine tumor aggressiveness
Source: Front Cell Dev Biol. 2022 Nov 18;10:1052098. doi: 10.3389/fcell.2022.1052098 (PMC9716026; doi:10.3389/fcell.2022.1052098)
Supplement: Supplementary file 5 [file Table3.docx]

**Supplementary Table 3. Comparison of immune cell infiltrates within metastatic tumors**

| **LM-uADC** | | | | |
| --- | --- | --- | --- | --- |
| Immune cell infiltrate | Tumor | ITF | Target tissue | p-value |
|  |  |  |  |  |
| CD20+ B lymphocytes | 17.7 ± 13.6 | 330.4 ± 256.2 | 6.2 ± 3.4 | 0.002** |
| CD4+ T cell | 234.2 ± 331.8 | 360.5 ± 421.5 | 134.1 ± 82.8 | 0.47 |
| CD8+ T cell | 322.2 ± 568.4 | 381.1 ± 486.5 | 66.6 ± 51.2 | 0.48 |
| CD68+ macrophages | 407.5 ± 522.4 | 515.9 ± 564.3 | 414.9 ± 358 | 0.6 |
| CD66+ neutrophils | 328.1 ± 665.8 | 145.5 ± 177.7 | 81.1 ± 80.6 | 0.7 |
|  |  |  |  |  |
| **LM-uLMS** | | | | |
| Immune cell infiltrate | Tumor | ITF | Target tissue | p-value |
|  |  |  |  |  |
| CD20+ B lymphocytes | 4.2 ± 8.3 | 21.6 ± 21.9 | 11.7 ± 17.2 | 0.012* |
| CD4+ T cell | 129.1 ± 196.4 | 97.2 ± 73 | 91.5 ± 110 | 0.85 |
| CD8+ T cell | 102.6 ± 139.7 | 102.7 ± 100 | 92.1 ± 73.4 | 0.76 |
| CD68+ macrophages | 238.8 ± 395.4 | 226.6 ± 134.2 | 375.0 ± 314.9 | 0.56 |
| CD66+ neutrophils | 19.9 ± 32.7 | 31.8 ± 22.7 | 26.2 ± 23.4 | 0.12 |

Quantification of immune cell types in the tumor area, invasive front (ITF) and lung (TT) within LM-uADC and LM-uLMS in 1x1 mm ROIs. Statistically significant differences in cells/mm^2^ are shown (p values, * <0.05, **<0.01). LM: tumor lung metastasis.
