# Supplementary material for: Integrating digital pathology with transcriptomic and epigenomic tools for predicting metastatic uterine tumor aggressiveness
Source: Front Cell Dev Biol. 2022 Nov 18;10:1052098. doi: 10.3389/fcell.2022.1052098 (PMC9716026; doi:10.3389/fcell.2022.1052098)
Supplement: Supplementary file 6 [file DataSheet2.docx]

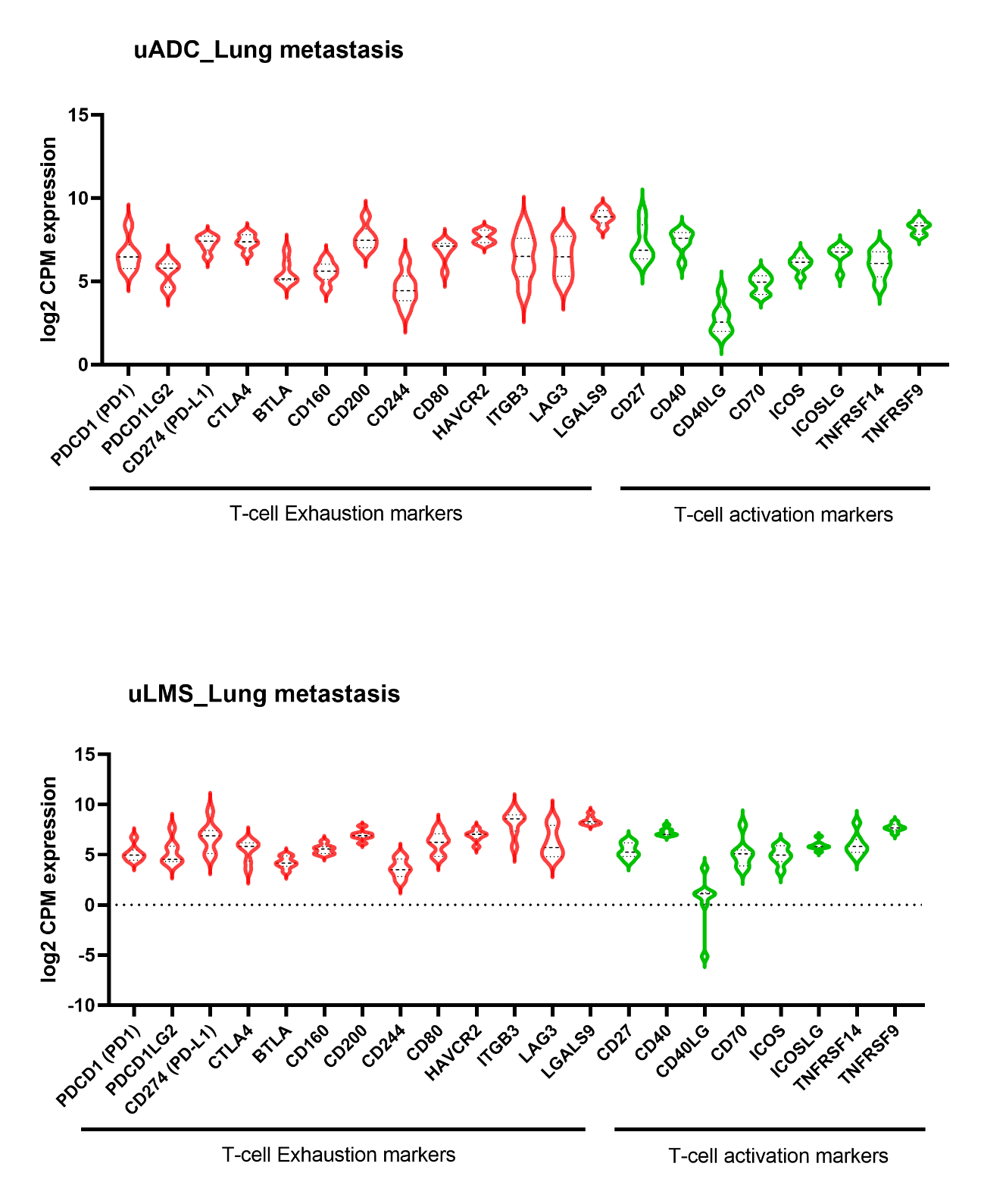


**Supplementary Figure 2.** Expression levels (RNA-seq, inmuno-oncoloy panel) of T-cell exhaustion and activation markers in the ITF of uADC and uLMS metastatic specimens.
