# Supplementary material for: Integrating digital pathology with transcriptomic and epigenomic tools for predicting metastatic uterine tumor aggressiveness
Source: Front Cell Dev Biol. 2022 Nov 18;10:1052098. doi: 10.3389/fcell.2022.1052098 (PMC9716026; doi:10.3389/fcell.2022.1052098)
Supplement: Supplementary file 8 [file DataSheet1.docx]

**
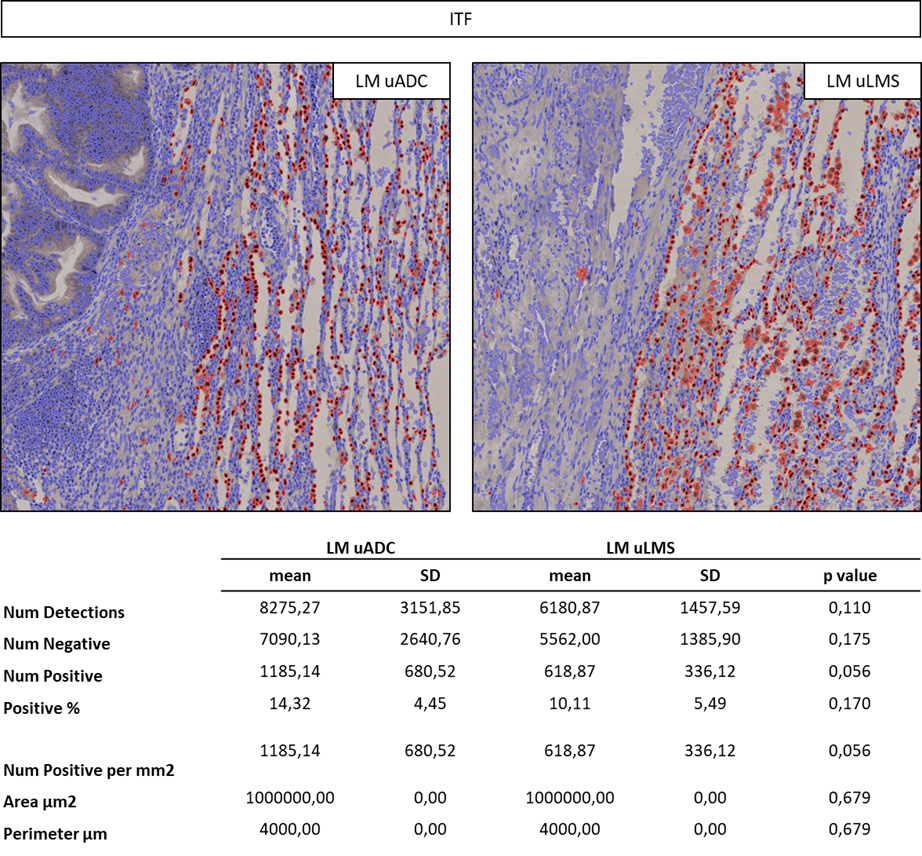
**

**Supplementary Figure 1. Comparison between the percentage (%) of Thyroid transcription factor 1 (TTF1) positive cells in LM-uADC and LM-uLMS at their ITFs.** Nucleus expressing TTF1 were detected with the Positive Cell Detection algorithm in QuPath. Cells marked in red represent TTF1-positive cells, while cells marked in blue represent TTF1-negative cells.
